# Staging of lymphoma under chimeric antigen receptor T-cell therapy: reasons for discordance among imaging response criteria

**DOI:** 10.1186/s40644-023-00566-7

**Published:** 2023-05-15

**Authors:** Michael Winkelmann, Viktoria Blumenberg, Kai Rejeski, Veit L. Bücklein, Maria Ingenerf, Marcus Unterrainer, Christian Schmidt, Franziska J. Dekorsy, Peter Bartenstein, Jens Ricke, Michael von Bergwelt-Baildon, Marion Subklewe, Wolfgang G. Kunz

**Affiliations:** 1 Department of Radiology, University Hospital, LMU Munich, Marchioninistr. 15, 81377 Munich, Germany; 2 Laboratory for Translational Cancer Immunology, Gene Center of the LMU Munich, Munich, Germany; 3 German Cancer Consortium (DKTK) and Bavarian Center for Cancer Research (BZKF), partner site Munich, Munich, Germany; 4 Department of Medicine III, University Hospital, LMU Munich, Munich, Germany; 5 Department of Nuclear Medicine, University Hospital, LMU Munich, Munich, Germany; 6 Comprehensive Cancer Center München-LMU (CCCM LMU ), LMU Munich, Munich, Germany

**Keywords:** CAR T-cell therapy, ^18^F-FDG PET/CT, Lugano criteria, Cheson, RECIL, LYRIC

## Abstract

**Background:**

Chimeric antigen receptor T-cell therapy (CART) prolongs survival for patients with refractory or relapsed lymphoma. Discrepancies among different response criteria for lymphoma under CART were recently shown. Our objective was to evaluate reasons for discordance among different response criteria and their relation to overall survival.

**Methods:**

Consecutive patients with baseline and follow-up imaging at 30 (FU1) and 90 days (FU2) after CART were included. Overall response was determined based on Lugano, Cheson, response evaluation criteria in lymphoma (RECIL) and lymphoma response to immunomodulatory therapy criteria (LYRIC). Overall response rate (ORR) and rates of progressive disease (PD) were determined. For each criterion reasons for PD were analyzed in detail.

**Results:**

41 patients were included. ORR was 68%, 68%, 63%, and 68% at FU2 by Lugano, Cheson, RECIL, and LYRIC, respectively. PD rates differed among criteria with 32% by Lugano, 27% by Cheson, 17% by RECIL, and 17% by LYRIC. Dominant reasons for PD according to Lugano were target lesion (TL) progression (84.6%), new appearing lesions (NL; 53.8%), non-TL progression (27.3%), and progressive metabolic disease (PMD; 15.4%). Deviations among the criteria for defining PD were largely explained by PMD of preexisting lesions that are defined as PD only by Lugano and non-TL progression, which is not defined as PD by RECIL and in some cases classified as indeterminate response by LYRIC.

**Conclusions:**

Following CART, lymphoma response criteria show differences in imaging endpoints, especially in defining PD. The response criteria must be considered when interpreting imaging endpoints and outcomes from clinical trials.

**Supplementary Information:**

The online version contains supplementary material available at 10.1186/s40644-023-00566-7.

## Background

Chimeric antigen receptor T cell therapy (CART) has emerged as an effective cell-based immunotherapy using patient-derived T cells targeting tumor antigens [1]. As a main application, the modified CAR T cells are used for the treatment of relapsed or refractory (r/r) lymphoma [2] and leukemia [3] with expression of CD19 antigen specific receptor. This has led to high rates of durable responses in large B-cell lymphoma (LBCL) (LBCL) [2; 4; 5], follicular lymphoma (FL) [2; 5], mantle-cell lymphoma (MCL) [6]. For initial staging and response assessment in the course of therapy, ^18^ F-fluorodeoxyglucose (^18^ F-FDG) positron emission tomography-computed tomography (PET/CT) is most commonly used.

In the currently ongoing phase III trials, treatment response is assessed using the Lugano criteria from 2014 [7; 8], which were established for lymphomas treated with conventional therapy. The Lugano criteria evolved from the Cheson criteria from 2007, which were used for response evaluation in previous studies [9]. In the recent years, new imaging criteria for lymphoma have been published. The two most important criteria in this regard are the lymphoma response to immunomodulatory therapy criteria (LYRIC) [10] from 2016 and the Response Evaluation Criteria for Lymphoma (RECIL) [11] from 2017. These were developed to better reflect the effects in the context of immunotherapies and, in part, to facilitate the measurement method [10; 11].

The scientific literature on structured comparisons of these imaging response criteria is scarce for conventional lymphoma treatments and only two studies indicate concordance of RECIL and Lugano criteria in previously untreated LBCL and FL [12; 13]. Recently, differences in OS stratification and median PFS among different response assessment criteria in lymphoma under CART were shown [14]. As there are no further reports on survival outcomes and the prognostic value for lymphoma patients treated with CART, we investigated the reasons for discordance among different imaging response criteria.

## Methods

### Study design and population

The study population was based on a prospective registry of all consecutive patients who were treated at the Comprehensive Cancer Center Munich-Ludwig-Maximilian University Munich (CCCM^LMU^) with standard-of-care CD19-specific CART products in between 01/2019 and 02/2022 (data cutoff). The following inclusion criteria were applied:

1. Patients with r/r lymphoma (LBCL, FL and MCL).
2. Any measurable disease on imaging according to Lugano criteria [7].
3. Available CT or PET-CT imaging studies at baseline (≤ 2 weeks before CART) and at least two follow-up timepoints (FU1 around 30 days and FU2 around 90 days, or before if clinical progression was evident).

The following exclusion criteria were applied:

1. Any non-diagnostic imaging studies.
2. Patients with non-measurable disease.
3. Lack of follow-up regarding survival data.

Histologic diagnoses were reviewed by expert pathologists. Patients received lymphodepletion with fludarabine and cyclophosphamide according to the manufacturers’ instructions.

### ^18^F-FDG PET/CT imaging

PET/CT images were acquired approximately 45 min after tracer injection (159–275 MBq weight-adapted with approximately 2.5–4.5 MBq ^18^ F-FDG per kg bodyweight) and for the FDG PET/CT contrast-enhanced or unenhanced CTs using a slice thickness of 2 mm 120 kVp, 100–400 mAs, and dose modulations were performed for attenuation correction. The following scanners were used: Biograph 64 and Biograph mCT (Siemens Healthineers, Germany) or Discovery 690 (GE Healthcare, USA). Both scanners fulfilled the requirements indicated in the European Association of Nuclear Medicine (EANM) imaging guidelines and obtained EANM Research Ltd. (EARL1) accreditation during acquisition. The following reconstruction algorithms were used: Biograph 64: TrueX (3 iterations, 21 subsets) with Gaussian post-reconstruction smoothing (2 mm full width at half-maximum). Biograph mCT: TrueX (3 iterations, 21 subsets). Discovery 690: VUE Point Fx algorithm with 2 iterations and 36 subsets. All systems resulted in a PET image with a voxel size of 2 × 2 × 2 mm3. Images were normalized to decay corrected injected activity per kg body weight (SUV g/ml).

### Imaging response assessment

To evaluate overall response, the Lugano criteria were applied and up to 6 target lesions (TL) were manually segmented by consensus of two radiologists with at least 5 years of experience in radiology and nuclear medicine. The sum of the product of the diameters (SPD) was measured to determine tumor burden (TB) for Lugano criteria, Cheson criteria, and LYRIC. In addition, spleen size was measured and splenomegaly defined by a vertical length > 13.0 cm according to Lugano criteria. Additional response criteria were applied to compare the overall response status. For Cheson criteria and LYRIC the same TL (≤ 6) as for Lugano criteria were evaluated. To assess response according to RECIL, the sum of longest diameters (SLD) of ≤ 3 TL was measured to define tumor burden. Depth of response (DoR) according to Lugano criteria, Cheson criteria, and LYRIC was calculated as the percentage decrease or increase in SPD from BL to FU2. DoR per RECIL was computed as the percentage change in SLD from BL to FU2.

Target lesions (TL), non-target lesions (NTL), and new appearing lesions (NL) in the course of therapy were evaluated quantitatively and qualitatively. The reason for progressive disease (PD) was analyzed for each response criterion and classified in the following categories: target lesion progression (TL PD), non-target lesion progression (NTL PD), appearance of new lesion(s) (NL PD), and progressive metabolic disease (PMD). The patient group with TL PD was divided in the 3 subgroups: progression of a single TL (uni), progression of up to 50% of the TL (oligo) or progression of ≥ 50% of the TL (multi).

We aligned our efficacy reporting standards with the Trial Reporting in Immuno-Oncology (TRIO) consensus statement by the American Society of Clinical Oncology (ASCO) and the Society of Immunotherapy of Cancer (SITC) [15]. All imaging analyses were performed with dedicated trial reporting software mintLesion 3.8 (mint Medical GmbH; Heidelberg, Germany). Organ distribution for TL, NTL and NL was documented and sub-grouped as nodal lesions and extranodal lesions.

### Statistical analysis

All statistical analyses were performed using GraphPad Prism 9. For survival analysis, OS was visualized using Kaplan-Meier survival curves with categorization for the patients to the response categories complete response (CR), partial response (PR), stable disease (SD), and progressive disease (PD) for all response criteria. The additional category of minor response (MR) was added for RECIL and indeterminate response (IR) for LYRIC. The overall response rate (ORR) was calculated as the rate of patients with CR and PR. Log-rank (Mantel-Cox) test was performed to examine the significance of the results. P values below 0.05 were considered to indicate statistical significance.

## Results

### Patient characteristics

**Fig. 1:**
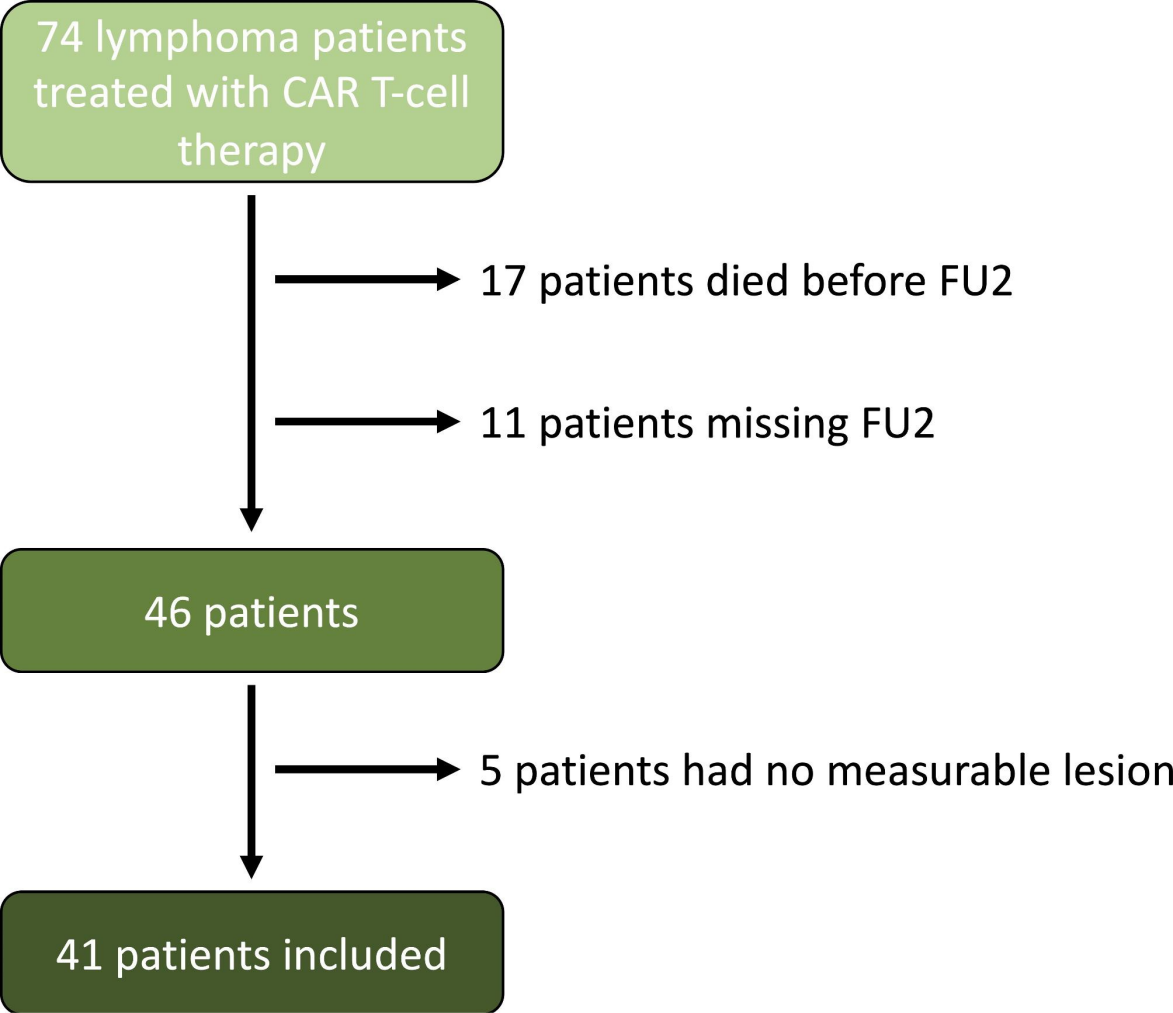
Flow Chart. A total of 74 lymphoma patients were treated with CAR T-cell therapy at our site in between 01/2019 and 02/2022. 17 patients who died before reaching FU2 and 11 patients without complete FU2 examination were excluded. 5 patients had no measurable lesion according to the Lugano criteria were also excluded. Finally, 41 patients met the inclusion criteria

Forty-one out of 74 patients met the inclusion criteria (median age: 64 years, 41% female). A flow chart is provided in Fig. 1. International prognostic index (IPI) was determined for all patients. IPI score 1 was present in 7 patients (17.1%), score 2 in 13 patients (31.7%), score 3 in 13 (31.7%), score 4 in 4 patients (9.8%), and score 5 in 4 patients (9.8%). 6 patients (14.6%) had stage I disease, 6 patients (14.6%) stage II, 7 patients (17.1%) stage III, and 22 patients (53.7%) stage IV according to Ann Arbor staging system. 31 out of 41 patients (75.6%) received a bridging therapy between apheresis and CAR T-cell infusion. Patient characteristics are shown in Table 1. Median TB at baseline was measured with a SPD of 4,835 mm^2^ and a SLD of 14.0 cm. Tumor burden, PFS and its correlation to OS are shown in Table 2.

**Table 1:** Patient Characteristics

Age (median)		64
**Gender**	Female: Male:	17 (41%) 24 (59%)
**Lymphoma entity**	LBCL: MCL: tFL:	35 (85%) 5 (12%) 1 (2%)
**Ann Arbor Stage**	I: II: III: IV:	6 (15%) 6 (15%) 7 (17%) 22 (54%)
**IPI**	1: 2: 3: 4: 5:	7 (17%) 13 (32%) 13 (32%) 4 (10%) 4 (10%)
**CAR T Product**	Tisagenlecleucel: Axicabtagene ciloleucel: Brexucabtagene autoleucel: Lisocabtagene maraleucel:	20 (58,8%) 13 (14,7%) 5 (23,5%) 3 (2,9%)
**Bridging**	Chemotherapy: Radiation: Immunotherapy: Combined therapy: No bridging:	21 (51%) 5 (12%) 2 (5%) 3 (7%) 10 (24%)
**LDH (median)**	Apheresis: Prior Lymphodepletion:	343 U/L 277 U/L

CAR, chimeric antigen receptor; LBCL, large B cell lymphoma; IPI, International Prognostic Index; LDH, lactate dehydrogenase; MCL, mantle cell lymphoma; tFL, transformed follicular lymphoma

**Table 2:** PFS, OS Association, Absolute and Relative DoR at 30d and 90d According to Criteria

Criteria	Baseline TB	FU1 TB	FU2 TB	Median PFS	PFS-OS Association	DoR FU1 (30d)	DoR FU2 (90d)
Lugano	4,835 mm^2^	1,265 mm^2^	1,101 mm^2^	153 d	r = 0.499 p = 0.03	-60.5	-78.1%
						-2,284 mm^2^	-2,338 mm^2^
Cheson	4,835 mm^2^	1,265 mm^2^	1,101 mm^2^	169 d	r = 0.476 p = 0.03	-60.5	-78.1%
						-2,284 mm^2^	-2,338 mm^2^
RECIL	14.0 cm	7.5 cm	6.8 cm	198 d	r = 0.535 p = 0.01	-41.7	-48.4%
						-4.3 cm	-6.1 cm
LYRIC	4,835 mm^2^	1,265 mm^2^	1,101 mm^2^	200 d	r = 0.758 p < 0.001	-60.5%	-78,1%
						-2,284 mm^2^	-2,338 mm^2^

Patient characteristics showing tumor burden (TB) at baseline (BL), follow-up 1 (FU1), and FU2. Median progression-free survival (PFS) is shown in days (d). The association of PFS and OS is presented as Pearson’s r. Both absolute and percentage depth of response (DoR) are reported according to each response criterion

### Depth of response (DoR)

**Fig. 2:**
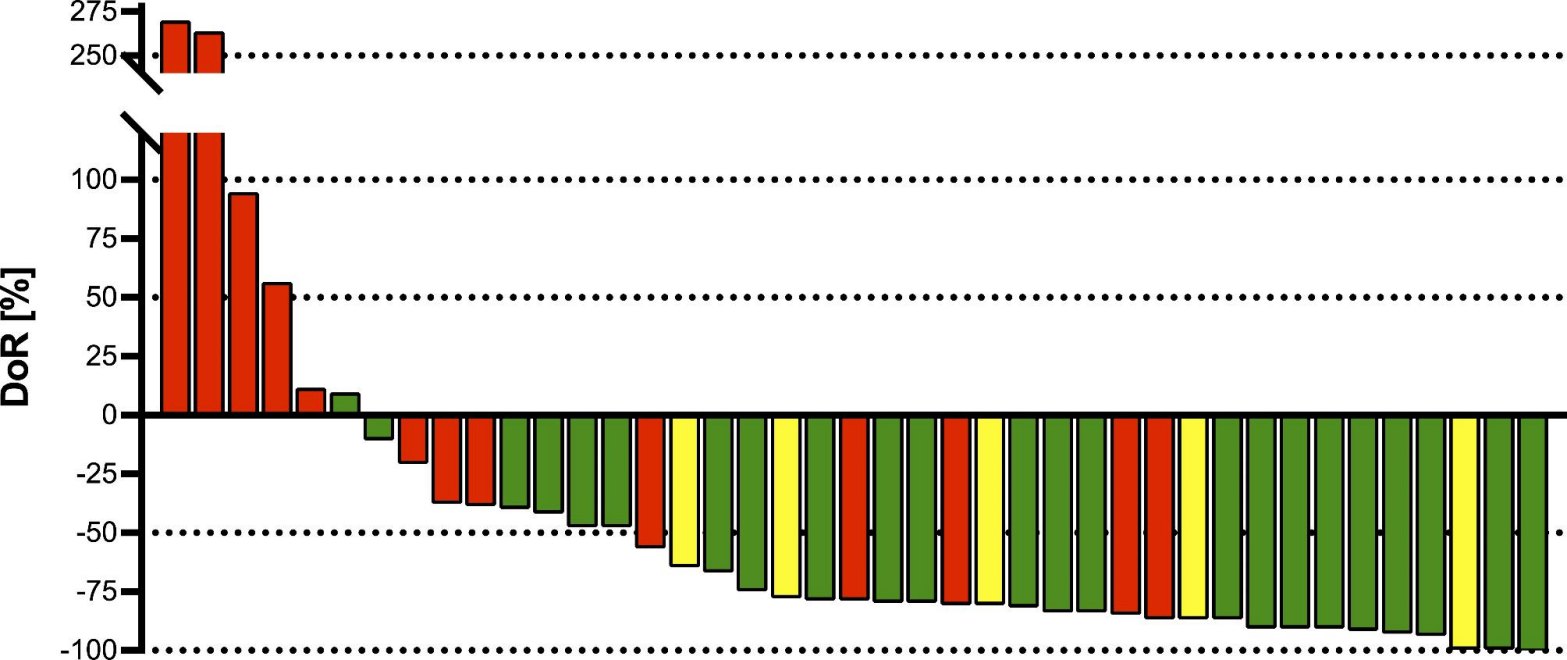
Target Lesion Change and Overall Response According to Lugano Criteria. Shown is a color-coded waterfall plot for depth of response (DoR) as percentage change of Lugano tumor burden (TB) of all 41 patients at FU2 (90d) compared to baseline. Positive values indicate an increase and negative values a decrease in TB. Bars are labeled red for progressive disease (PD), yellow for partial response (PR) and green for complete response (CR) at FU2 according to Lugano criteria

DoR at FU1 and FU2 was calculated for all response criteria and is shown in Table 2. At FU1, median SPD was 1,265 mm^2^ and SLD 7.5 cm. At FU2, TB decreased to a median SPD of 1,101 mm^2^ and SLD of 6.8 cm. According to RECIL, median depth of response (DoR) was − 4.3 cm (-41.7%) at FU1 and − 6.1 cm (-48.4%) at FU2. For all other response criteria median DoR was − 2,284 mm^2^ (-60.5%) at FU1 and − 2,338 mm^2^ (-78.1%) at FU2. DoR by Lugano criteria, Cheson criteria, and LYRIC as percent increase or decrease in SPD for all 41 patients is illustrated in Fig. 2. The color coding of the waterfall plot was chosen according to the categories of the Lugano criteria. Although most patients showed a good DoR, some of them only had a PR after 3 month or even a PD according to Lugano criteria, despite showing a significant decrease in TB.

### Overall response according to Lugano, Cheson, RECIL, and LYRIC

**Fig. 3:**
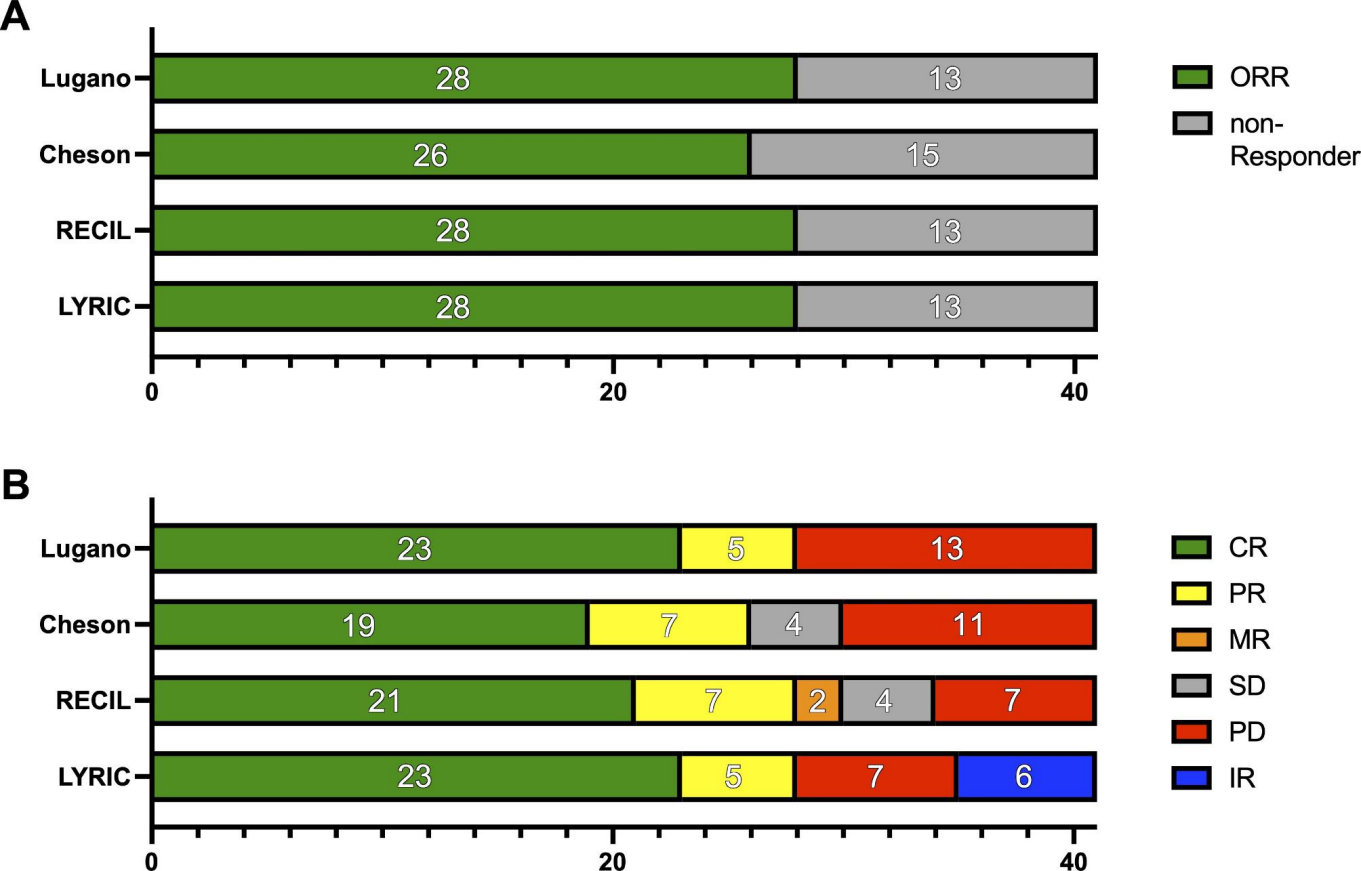
Overall Response According to Lugano, Cheson, RECIL, and LYRIC. The upper row (**A**) depicts the distribution of patients with overall response (ORR; green) and non-responders (gray) for different response criteria: Lugano, Cheson, response evaluation criteria for lymphoma (RECIL) and lymphoma response to immunomodulatory therapy criteria (LYRIC). In the lower part (**B**), the bar plot visualizes the number of patients allocated to the different response categories according to each criterion. Patients with complete response (CR) are labeled green, patients with partial response (PR) yellow, patients with stable disease (SD) gray, and patients with progressive disease (PD) red. For RECIL, patients with minor response (MR) are labeled orange. For LYRIC, patients with indeterminant response (IR) are labeled blue

The overall response rate (ORR) was comparable among the different criteria with 59%, 59%, 56%, and 59% at FU1 and 68%, 68%, 63%, and 68% at 90 day FU (FU2) according to Lugano, Cheson, RECIL, and LYRIC, respectively. Applying the Lugano criteria in FU2, 23 patients (56.1%) showed a CR and 5 patients (12.2%) showed a PR, whereas 13 patients (31.7%) had a PD. Discordance in the classification of overall response and rate of progressive patients was observed when other response criteria were applied (Fig. 3). Interestingly, Cheson criteria and RECIL classified 4 patients as a SD, whereas there were none according to Lugano criteria. In addition, RECIL classified 2 patients as minor response (MR) and LYRIC classified 6 patients as indeterminate response (IR).

### PFS and reasons for progression according to different criteria

**Fig. 4:**
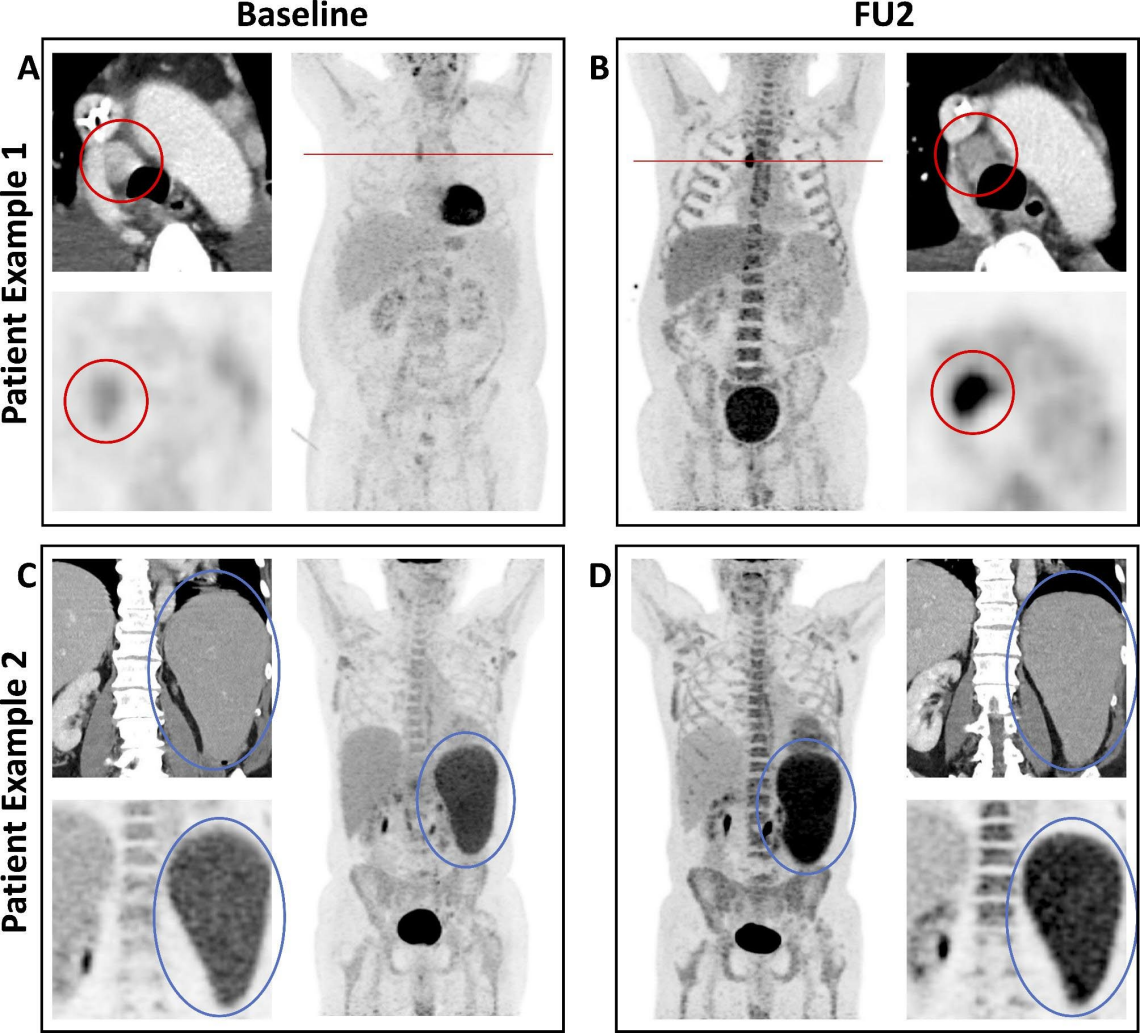
Patient Examples with Discrepancy Between Lugano, RECIL and LYRIC. PET/CT images of patient example 1 are illustrated in the upper panel with baseline scan on the left (**A**) and follow-up staging after 90 days (FU2) on the right (**B**). The patient had progressive metabolic disease of a mediastinal nodal lymphoma manifestation (red circle) without increase in size. All other TL and NTL showed significant DoR, and no NL appeared. This results in progressive disease according to Lugano criteria, but partial response by Cheson criteria and RECIL, and indeterminate response according to LYRIC. The second patient example is shown in the lower panel (**C** + **D**) with a progressive metabolic disease of the spleen without further increase in size. This was defined as PD by Lugano criteria and IR by LYRIC. The decrease in size of all other TLs indicated response according to RECIL (PR) and stable disease according to Cheson criteria

Median PFS_Lugano_ was 153 days, PFS_Cheson_ 169 days, PFS_RECIL_ 198 days, and PFS_LYRIC_ 200 days. The reason for progressive disease was analyzed for each response criterion, as shown in Table 3. The categories TL PD, NTL PD, NL PD, and PMD were applied as described above. Dominant reasons for PD according to Lugano criteria were target lesion (TL) progression as size increase of one or more TL (84.6%), appearance of new lesions (NL; 53.8%), non-TL progression (27.3%), and progressive metabolic disease (PMD, 15.4%). In most patients with progressive disease, there was a multifactorial cause for progression. According to Lugano criteria, 7/13 patients (53.8%), by Cheson criteria 6/11 patients (54.5%), by RECIL 4/7 patients (57.1%), and by LYRIC 6/7 patients (85.7%) had progression with multiple causes. An example of two patients with discordant response criteria is illustrated in Fig. 4. Deviations among the criteria for defining PD were largely explained by PMD of preexisting lesions that are defined as PD only by Lugano and non-TL progression, which is not defined as PD by RECIL and in some cases classified as IR by LYRIC.

**Table 3:** Reasons for Classification of Progressive Disease According to Criteria

Criteria	TL PD	Focality of Progressive Lesions			NTL PD	NL PD				New Organ Lesion	PMD
		Uni	Oligo	Multi		Total	Nodal	Extra-nodal	Both		
Lugano	10	4	1	5	3	7	2	1	4	Bone (3) Muscle (3) Peritoneal (2) Fat tissue Kidney Liver Lung Pancreas Pleura	2
Cheson	10	4	1	5	3	7	2	1	4		0
RECIL	7	1	1	5	0	7	2	1	4		0
LYRIC	6	0	1	5	1	6	1	1	4		0

Comparison of different imaging response criteria and the reasons for progressive disease. Shown are patient numbers with uni, oligo or multi target lesion (TL) progression, non-target lesion (NTL) progression, appearance of new lesions (NL) and progressive metabolic disease (PMD). Overlap of progression by multiple causes occurred in 53.8% of progressive patients by Lugano criteria, in 54.5% of patients by Cheson criteria, in 57.1% by RECIL, and 85.7% by LYRIC.

### Survival analysis and reasons for progression by different response Criteria

There was a significant difference in survival between patients who responded to therapy in FU2 with a CR or PR compared to patients who did not respond to therapy with MR, SD or PD. Classification of patients into these two groups allowed significant stratification of OS for all response criteria (Supplementary Fig. 1; p < 0.001), with those who responded having longer OS. For the new IR category introduced by LYRIC, we observed a non-significant difference compared to the non-responding group (Supplementary Fig. 1D; p = 0.224).

**Fig. 5:**
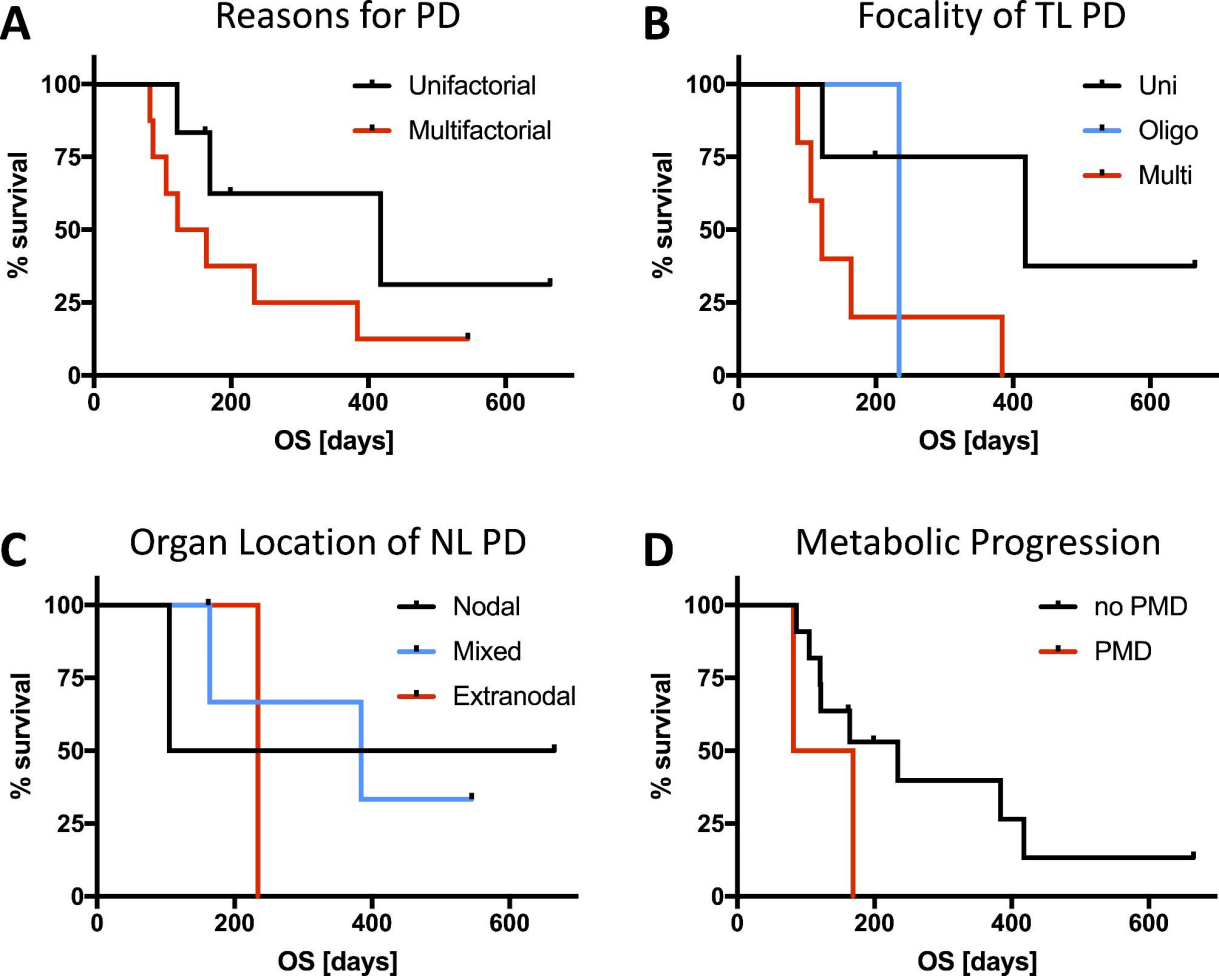
Overall Survival Stratification by Reason for Progression. Illustrated are the Kaplan-Meier survival curves for overall survival (OS) for the different reasons for progressive disease (PD) according to Lugano criteria. Patients with multifactorial causes for classification as PD had a non-significant difference in OS compared to patients with an unifactorial cause for PD (p = 0.185) as shown in **A**. Similar results were observed for metabolic progression (**D**) with a small yet non-significant difference between patients with progressive metabolic disease (PMD) and patients with no PMD (p = 0.192). Between the groups with uni, oligo, and multi target lesion (TL) PD (**B**), there was a significant stratification of OS (p = 0.036) with patients with unifocal TL progression having a longer OS. Grouping patients according to the location of new lesions (NL), either nodal only, extranodal or mixed showed no significant difference in OS (**C**; p = 0.700)

In a next step, we analyzed whether there was a difference in the OS for the main reasons for discordance between the respective criteria. As mentioned above and shown in Table 2, the main reasons for discordance were focality of TL PD, organ location of NL PD, metabolic progression (Lugano-based PMD vs. non-PMD), and whether the progression was unifactorial or multifactorial. There was a non-significant difference in OS for patients with multifactorial causes for PD compared to patients with an unifactorial cause (Fig. 5A; p = 0.185). Similar results could be observed for the location of the NL with the groups: nodal, extranodal, and mixed (Fig. 5C; p = 0.700), as well as metabolic progression according to Lugano criteria (Figs. 0 and 5D.192). A significant trend between the uni, oligo, and multi groups for location of TL PD (Fig. 5B; p = 0.036) was detected, with patients with progression of only one TL having a longer OS.

## Discussion

In this population of patients treated with CD19 CART, both the established and explorative lymphoma response criteria showed considerable discordance in imaging endpoints based on different reasons for definition of PD. While the ORR was almost unaffected, classification of patients as SD and PD differed significantly. In addition, some patients with the new proposed response categories MR by RECIL and IR by LYRIC were identified. Dichotomization into responding and non-responding patients based on 3-month FU stratified OS by all criteria. Interestingly, grouping patients based on the Lugano TL PD into groups with uni TL, oligo TL and multi TL progression showed a significant trend for OS stratification.

Lymphoma response criteria have historically been developed and established in the first-line treatment setting, and notably in an era of cytotoxic chemotherapies. The Cheson and Lugano criteria have evolved from the unidimensional Response Evaluation Criteria in Solid Tumors (RECIST1.1) criteria [16] and measure the bidimensional extension for this typically nodal-dominant tumor phenotype [7; 9]. On CT imaging, patients with pretreated lymphoma often have residual masses that can easily be mistaken as vital tumor [17]. Therefore, the imaging response criteria for lymphoma incorporate metabolic activity of lymphoma manifestations as visualized by ^18^ F-FDG PET/CT in order to identify a complete response [7; 9–11].

In the development of the response evaluation criteria in lymphoma (RECIL), the panelists set out to facilitate the response assessment of lymphoma by reducing the number of target lesions that need to be measured to achieve similar validity as the Lugano criteria [11]. A reduction to 3 target lesions to represent tumor burden enabled robust response classification compared to the other criteria that rely on 6 manifestations. In addition, RECIL require a combination of depth of response and reduction of metabolic activity to classify a response.

In the first-line treatment setting, the association of imaging endpoint surrogates of survival as PFS is known to be strong regarding OS [18; 19]. However, in the course of the disease with r/r lymphomas, changes in the phenotype and metabolism of the manifestations occur. Typically, distant lymph nodes are more commonly affected and extranodal lesions are more frequent. In addition, preexisting remnants may be mistaken for active lymphoma if previous imaging studies are not reviewed. Notably, response criteria have not been adjusted for such changes in disease progression, neither in solid nor hematologic malignancies, and data on the association of PFS and OS in lymphoma is scarce [7; 16].

In the context of CAR T-cell therapy data on characterization of response by classification system is very limited. A single-arm, prospective study of 7 patients with LBCL and FL, treated with CD19 CART evaluated early response according to Lugano criteria on 1-month ^18^ F-FDG PET/CT [20]. Interestingly all patients in this study with less than CR subsequently relapsed [20]. A multicenter study with 171 patients analyzed the Deauville score of NHL patients under CART in 1 month FU ^18^ F-FDG PET/CT with similar results. Patients with Deauville Score 1 + 2 at 1 month FU had an improved long-term outcome compared to patients with Deauville Score 3–5, who were at risk for an early relapse. Moreover, all patients with Deauville Score 5 relapsed by month 3 [21]. Another group suggested a SUVmax ≥ 10 at 1 month as a predictor of progression [22]. Recently, it was shown that pretreatment tumor burden metrics of lymphoma under CART vary significantly based on the assessment method, impacting their association with survival outcomes [23].

The analysis of specific response patterns of lymphoma and the impact of pseudoprogression in the context of CART, has not yet been studied in detail [24]. Pseudoprogression is defined by a transient increase in tumor size due to an infiltration of the tumor by immune cells and is mainly described in solid tumors under immunotherapy, particularly in melanoma, affecting 5–12% of cases [25]. Few studies described cases of pseudoprogression after CART analogous to solid tumors [26].

To prevent patients with pseudoprogression from being misclassified as progressive disease LYRIC introduced the category of indeterminate response (IR) with 3 subcategories: IR1, increase in SPD increase ≥ 50% within the first 12 weeks of therapy, without clinical deterioration; IR2, appearance of new lesions, or growth of one or more existing lesions ≥ 50% at any time during treatment in the absence of overall progression; IR3, increase in FDG uptake of one or more lesions without a concomitant increase in lesion size or number [10]. LYRIC suggests follow-up in all IR cases after 12 weeks and encourages a biopsy for IR1 and IR2. In contrast to LYRIC, the other response criteria do not provide recommendations for lesion follow-up [7; 9; 11].

Another feasible method for distinguishing pseudoprogression from true progression would be immuno-PET, which uses mAbs or antibody fragments radiolabeled with a positron emitter radionuclide that can be detected on PET/CT imaging [27]. For lymphoma patients, there are a variety of potential targets, such as T-cell markers (CD3, CD4, and CD8), B-cell markers (CD19 or CD20), and immune checkpoints (PD-1, PD-L1, or CTLA-4) [28]. The first clinical trial with 5 patients included demonstrated the suitability of immuno-PET with ^89^Zr-rituximab (anti-CD20 MAC) in patients with relapsed B-cell NHL [29]. In a later study, iPET with ^89^Zr-labeled anti-CD20 mAbs was suggested as a potential biomarker for predicting the response of r/r DLBCL [30]. Immuno-PET has also been used to visualize the migration, activation, and expansion of CD19-specific CAR-T cells in an in vivo mouse model of B-cell lymphoma [31]. However, there is very few clinical data, especially in the context of CART and limited availability of immune-PET in clinical routine.

In our study, there was no difference in terms of OS in patients with NL PD, even when sub-analyzed by the location of newly appearing lesions. In addition, patients with LYRIC-based IR had a nonsignificant difference in survival compared with patients with PD. Therefore, patients with solely newly appearing lesions and patients with LYRIC-based IR should be further investigated regarding clinical benefit and may represent a new response category. NL biopsy for histological workup should be considered in these cases. Alternatively, liquid biopsy using ctDNA may represent a minimally invasive test to resolve diagnostic uncertainties in this clinical scenario [32; 33].

In addition, LYRIC-based PFS showed the strongest association with OS. One explanation could be that LYRIC effectively classifies patients with pseudoprogression into one of the IR categories. Another explanation is that patients with a small increase in tumor burden or growth of a single TL are also classified as IR, in contrast to the Lugano or Cheson criteria, in which a single significantly growing TL is classified as PD. In these patients, the lymphoma may indeed progress, but perhaps with low kinetics, resulting in longer OS. This would be consistent with our findings that patients with single-site TL PD have a longer OS than patients with oligo- or multi-site TL PD. To address this issue, further characterization of tumor kinetics would be interesting. Recently, it has been shown that the increase in tumor growth rate post-baseline compared to pre-baseline in lymphomas treated with CART has a significant impact on OS [34].

Future response assessment in lymphoma with novel imaging endpoints and response criteria will likely be based on assessment of whole tumor burden (e.g. metabolic tumor volume) and not only based on selected lesions. In the first-line setting of LBCL, the recently published International Metabolic Prognostic Index (IMPI), that consists of metabolic tumor volume, age, and stage, has outperformed the conventional IPI in estimating outcome [35]. These imaging findings may be integrated with prognostic risk-stratification tools such as the CAR-HEMATOTOX [36; 37]. Further areas of study may also focus on patterns of response, e.g. volume changes or the absolute number and size of new lesions. Such criteria refinements have been successfully applied in other cancer entities in the advanced, later-line disease stage, for example in metastatic prostate cancer [38; 39].

Recently, differences in imaging endpoints among response criteria in lymphoma were reported [14]. To our knowledge, there is no further literature comparing the response assessment in the context of r/r lymphoma under CART. Our study has limitations which need to be considered when interpreting the results. First, this is a single-center study with a limited number of subjects. Second, there were a few patients that were missed to follow-up or had no measurable disease. Not all patients had a PET-based assessment at day 30.

## Conclusions

We investigated overall response by Lugano criteria, Cheson criteria, RECIL, and LYRIC. While the ORR was comparable between the different criteria, considerable discordances in imaging endpoints based on different reasons for definition of PD. Response assessment by LYRIC exhibited superior association between PFS and OS. In addition, we could detect a significant trend for OS stratification by grouping the patients into the 3 groups: uni, oligo, and multi TL PD. The response assessment method must therefore be considered when interpreting the impact of imaging endpoints on outcomes in clinical trials. Considering the heterogeneity, our results argue for standardization and harmonization across centers.

## Electronic supplementary material

**Additional File 1** Supplementary Figure 1. Overall Survival of Different Criteria According to Response

## Data Availability

The datasets generated during and/or analysed during the current study are available from the corresponding author on reasonable request.

## List of abbreviations

CART: Chimeric antigen receptor T-cell therapy
CR: Complete response
DoR: Depth of response
FDG: Fluorodeoxyglucose
FL: Follicular lymphoma
FU: Follow-up
IPI: International prognostic index
IR: Indeterminate response
LBCL: Large B-cell lymphoma
LYRIC: Lymphoma response to immunomodulatory therapy criteria
MCL: Mantle-cell lymphoma
MR: Minor response
NL: New appearing lesions
NTL: Non-target lesion
ORR: Overall response rate
OS: Overall survival
PET/CT: Positron emission tomography-computed tomography
PD: Progressive disease
PFS: Progression-free survival
PMD: Progressive metabolic disease
PR: Partial response
r/r: Relapsed or refractory
RECIL: Response evaluation criteria in lymphoma
SD: Stable disease
SLD: Sum of longest diameters
SPD: Sum of the product of the diameters
TB: Tumor burden
TL: Target lesion
