# Supplementary material for: Staging of lymphoma under chimeric antigen receptor T-cell therapy: reasons for discordance among imaging response criteria
Source: Cancer Imaging. 2023 May 15;23:44. doi: 10.1186/s40644-023-00566-7 (PMC10184388; doi:10.1186/s40644-023-00566-7)
Supplement: Supplementary file 1 — Additional File 1 Supplementary Figure 1. Overall Survival of Different Criteria According to Response [file 40644_2023_566_MOESM1_ESM.docx]

**SUPPLEMENT**

**Supplementary Figure 1.** Overall Survival of Different Criteria According to Response


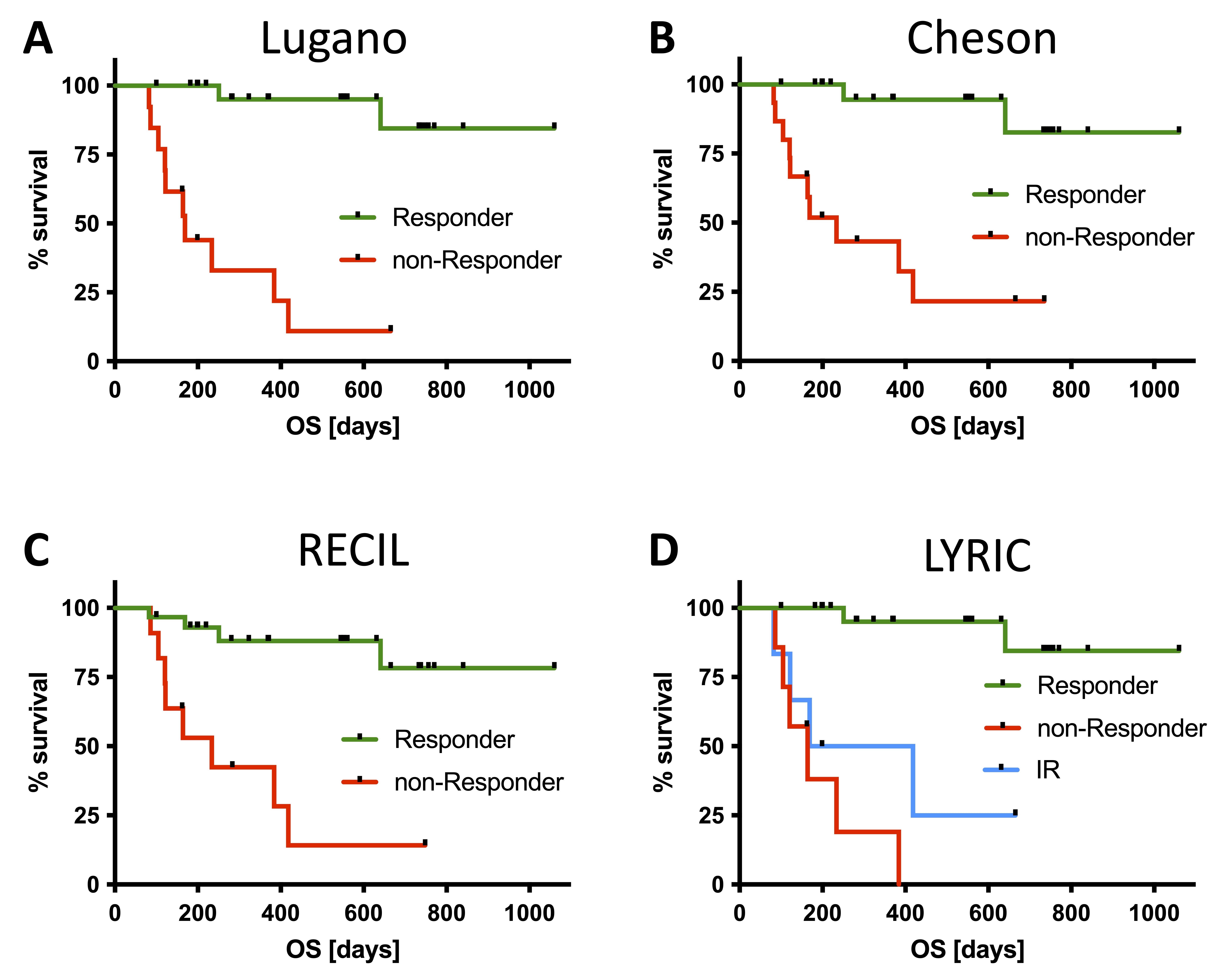
Depicted are the Kaplan-Meier survival curves for overall survival (OS) with grouping into responding (green) and non-responding (red) patients. For Lugano criteria (**A**), Cheson criteria (**B**), response evaluation criteria for lymphoma (RECIL; **C**), and lymphoma response to immunomodulatory therapy criteria (LYRIC; **D**) show a significant difference in OS for the two groups (p<0.001). For the indeterminant response (IR) category (blue), newly introduced by LYRIC, there is a non-significant difference between responding to non-responding patients (**D**; p=0.224).
